# *Achillea millefolium* L. Essential Oil Inhibits LPS-Induced Oxidative Stress and Nitric Oxide Production in RAW 264.7 Macrophages

**DOI:** 10.3390/ijms140712978

**Published:** 2013-06-24

**Authors:** Su-Tze Chou, Hsin-Yi Peng, Jaw-Cherng Hsu, Chih-Chien Lin, Ying Shih

**Affiliations:** 1 Department of Food and Nutrition, Providence University, 200, Sec. 7, Taiwan Boulevard, Shalu Dist., Taichung 43301, Taiwan; E-Mails: stchou@pu.edu.tw (S.-T.C.); g9522501@pu.edu.tw (H.-Y.P.); 2 Department of Applied Cosmetology, Hung Kuang University, 1018, Sec. 6, Taiwan Boulevard, Shalu Dist., Taichung 43302, Taiwan; E-Mail: jchsu@sunrise.hk.edu.tw; 3 Department of Cosmetic Science, Providence University, 200, Sec. 7, Taiwan Boulevard, Shalu Dist., Taichung 43301, Taiwan

**Keywords:** *Achillea millefolium* L., antioxidant, anti-inflammatory, essential oil, gas chromatography-mass spectrometry (GC-MS), lipopolysaccharides (LPS)-stimulated RAW 264.7 macrophages

## Abstract

*Achillea millefolium* L. is a member of the Asteraceae family and has been used in folk medicine in many countries. In this study, 19 compounds in *A. millefolium* essential oil (AM-EO) have been identified; the major components are artemisia ketone (14.92%), camphor (11.64%), linalyl acetate (11.51%) and 1,8-cineole (10.15%). AM-EO can suppress the inflammatory responses of lipopolysaccharides (LPS)-stimulated RAW 264.7 macrophages, including decreased levels of cellular nitric oxide (NO) and superoxide anion production, lipid peroxidation and glutathione (GSH) concentration. This antioxidant activity is not a result of increased superoxide dismutase (SOD), catalase (CAT), glutathione peroxidase (GPx) activities, but rather occurs as a result of the down-regulation of inducible nitric oxide synthase (iNOS), cyclooxygenase-2 (COX-2), tumor necrosis factor-α (TNF-α), interleukin-6 (IL-6) and heme oxygenase-1 (HO-1) expression, thus reducing the inflammatory response. Therefore, AM-EO can be utilized in many applications, including the treatment of inflammatory diseases in the future.

## 1. Introduction

Inflammatory response, a physiological reaction to infection or damage, plays a significant role in health and disease [1]. Macrophage has a significant impact in immune response and inflammation. The cells inducing inflammation, also initiate and maintain specific immune responses by secreting various types of cytokines [2]. Lipopolysaccharides (LPS), a toxic molecule derived from gram-negative bacteria cell walls, activates macrophages to release numerous inflammatory mediators, such as nitric oxide (NO), superoxide anion, cyclooxygenase (COX)-2, tumor necrosis factor-α (TNF-α) and interleukin-6 (IL-6) [3]. Over production of these inflammatory mediators is involved in many inflammation associated disorders. Therefore, inflammatory mediator inhibition is believed to be a good approach for treatment of inflammatory diseases [4].

*Achillea millefolium* L., known as yarrow, is a member of the Asteraceae family and has been used in folk medicine for hundreds of years in many countries ranging from Europe to Asia [5]. Yarrow has been traditionally used to treat inflammatory and spasmodic gastrointestinal disorders, hepatobiliary complaints and overactive cardiovascular and respiratory ailments [6,7]. In addition, yarrow is used as an appetite-enhancing agent because of its bitter taste; it has further been used in wound healing, as an antiulcer agent and as an anti-inflammation agent [7,8].

The aerial parts of *A. millefolium* are generally applied as aqueous or alcoholic extracts. Many studies have reported antioxidant and anti-inflammatory activities [9–11], antimicrobial and antifungal activities [10,12], gastroprotective properties [13] and estrogenic activity [14] of *A. millefolium* extracts. Moreover, the extracts of *A. millefolium* exhibit potent anticancer activity in several types of tumor cells, including leukemia [15], cervical and breast epithelial adenocarcinoma, skin epidermoid carcinoma [16], hepatoma [17] and lung tumor cells [18]. Thus, extracts from this plant may have potential to be applied in several fields, including medicine, food additives and cosmetics.

In addition, research on the phytochemical composition of *A. millefolium* has shown that it contains an abundance of phenolic compounds, such as flavonoids and phenolic acids [5,7]. Furthermore, some—but not enough—studies have revealed the chemical components of its essential oil [19–21]. However, until now, the complete characteristics and biological functions of the essential oil of *A. millefolium* remain unknown. Therefore, in this study, we analyzed its chemical compositions using gas chromatography-mass spectrometry (GC-MS) to identify the functional components of *A. millefolium* essential oil (AM-EO). The relationships between antioxidant and anti-inflammatory activities of AM-EO were examined *in vitro*. Moreover, the anti-inflammatory mechanism of AM-EO in LPS-induced murine macrophage cells was also investigated in the study.

## 2. Results and Discussion

### 2.1. Chemical Composition of AM-EO

**Figure 1:**
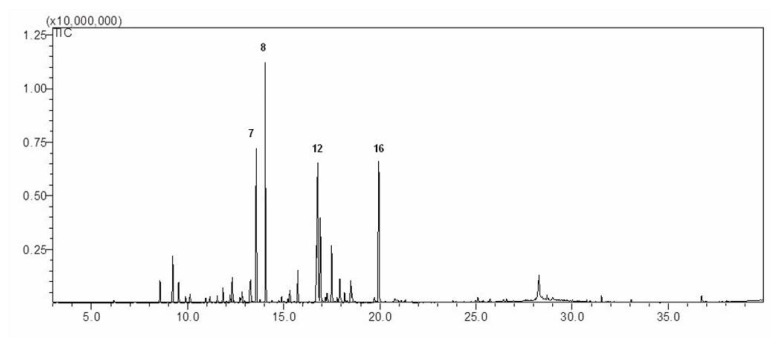
Total ion chromatogram of *Achillea millefolium* L. essential oil from gas chromatography-mass spectrometry (GC-MS).

Steam-distilled essential oil of AM-EO was purchased from Australian Botanical Products, Pty Ltd. (Hallam, Victoria, Australia) and then analyzed with GC-MS. In AM-EO, 19 primary compounds were identified and are listed in Table 1, in addition to the retention times and Kovats indices. The total ion chromatogram of AM-EO is shown in Figure 1.

The results show that the most abundant constituent of AM-EO is artemisia ketone (14.92%). Other major components of AM-EO include camphor (11.64%), linalyl acetate (11.51%) and 1,8-cineole (10.15%). In addition, AM-EO contains D-limonene (7.39%), linalool (6.55%), yomogi alcohol (6.36%), borneol (5.37%) and o-cymene (5.26%). The most abundant components within AM-EO are monoterpene hydrocarbons and oxygen monoterpenes, which comprise 18.00% and 75.72% of the total, respectively. AM-EO also is composed of 4.59% sesquiterpene hydrocarbons (Table 1).

Previous studies that have determined the chemical composition of *A. millefolium* essential oils also identified high levels of artemisia ketone (4.1% to 12.6%), camphor (6.1% to 24.5%), 1,8-cineole (11.4% to 40.4%), linalool (0.9% to 9.5%) and borneol (3.2% to 9.2%) [19–21]. However, linalyl acetate was only found in trace amounts in other studies of *A. millefolium* essential oil; this difference might be due to the diversity of the plant sources or different essential oil hydrodistillation procedures. Moreover, earlier studies have shown that high quantities of monoterpene hydrocarbons and oxygen sesquiterpenes are present in *A. millefolium* essential oils, ranging from 10.4% to 26.9% monoterpene hydrocarbons and 20.8% to 78.4% oxygen monoterpenes [19]. Previous studies also indicated that the essential oils with high levels of artemisia ketone, camphor, 1,8-cineole, linalool and borneol frequently feature some important biological functions, such as antioxidant, anti-inflammatory, antimicrobial and anticancer activities [10,21–24]. In addition, anti-inflammatory activity of the AM-EO major components, camphor (11.64%), linalyl acetate (11.51%) and 1,8-cineole (10.15%), have been demonstrated by several earlier studies [25–29]. Therefore, it can be proposed that the oil examined might also exhibit antioxidant and anti-inflammatory activities, suggesting that its biological functions and mechanisms should be studied further.

### 2.2. Effects of AM-EO on Cell Viability and NO Production

**Figure 2:**
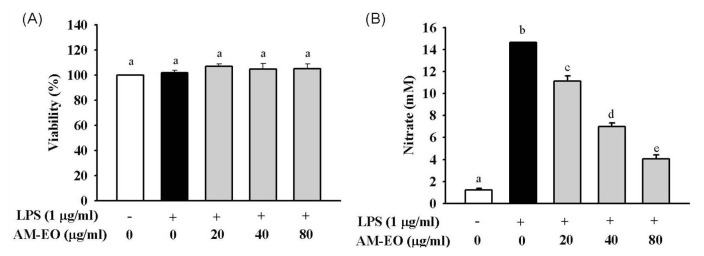
The effect of *A. millefolium* essential oil (AM-EO) on (**A**) cell viability and (**B**) nitric oxide (NO) production in lipopolysaccharides (LPS)-induced RAW 264.7 macrophages. Each value represents the mean ± SD (*n* = 3). Groups sharing the same superscript letter are not significantly different (*p* > 0.05) as revealed by Dunnett’s *post hoc* tests.

To evaluate the effects of AM-EO on LPS-stimulated (l μg/mL) RAW 264.7 macrophages and to determine the optimal concentrations for the following analyses, a standard MTT assay was used to test the effect of AM-EO on cell viability. The results are shown in Figure 2A. All tested concentrations (from 20 to 80 μg/mL) of AM-EO did not decrease the cell viability of LPS-stimulated RAW 264.7 macrophages. Additionally, AM-EO treatment was found to slightly increase the number of LPS-stimulated RAW 264.7 macrophages (Figure 2A).

LPS-stimulated RAW 264.7 macrophages are expected to produce NO because NO is a toxic molecule released by the innate immune cells during pathogenesis. As shown in Figure 2B, AM-EO treatment decreased NO production in LPS-stimulated RAW 264.7 macrophages at all concentrations tested (20, 40 and 80 μg/mL). AM-EO treatment at 80 μg/mL can reduce NO production by approximately 35% (Figure 2B), and this inhibition by AM-EO occurs in a dose-dependent manner. These results indicate that AM-EO has no cytotoxicity in any of the tested concentrations and may decrease NO production in LPS-stimulated RAW 264.7 macrophages.

### 2.3. The Effect of AM-EO on Superoxide Anion and Malondialdehyde (MDA) Production, GSH Concentration and LPS-Induced DNA Damage

To confirm that the anti-inflammatory activity of AM-EO in LPS-stimulated RAW 264.7 macrophages was due to its antioxidant property, we investigated some critical indicators of oxidative stress from the cells treated with AM-EO.

**Figure 3:**
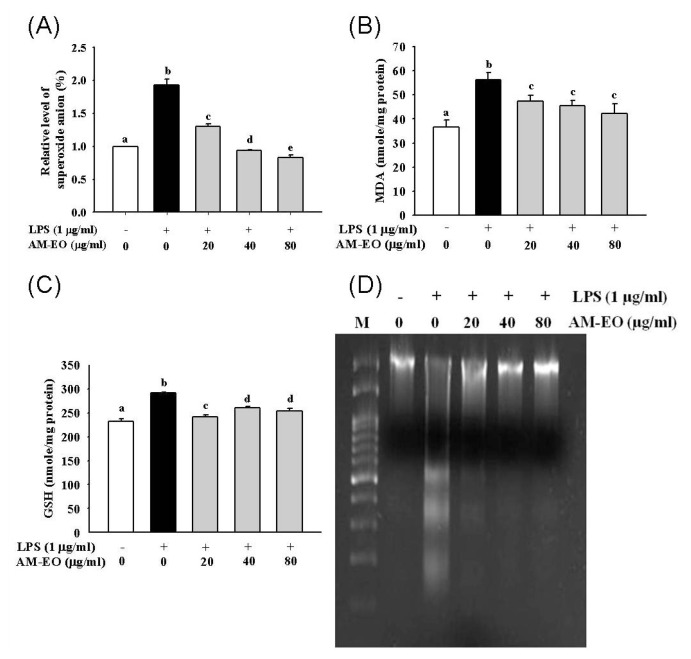
The effect of AM-EO on (**A**) superoxide anion, (**B**) malondialdehyde (MDA) production, (**C**) glutathione (GSH) concentration and (**D**) DNA damage by LPS-induced RAW 264.7 macrophages. Each value represents the mean ± SD (*n* = 3). Groups sharing the same superscript letter are not significantly different (*p* > 0.05) as revealed by Dunnett’s *post hoc* tests.

First, superoxide anion production was analyzed in AM-EO-treated cells, and the results are shown in Figure 3A. Compared to the LPS-treated cells, superoxide anion production in the AM-EO-treated cells decreased by 58% at concentrations of 20 μg/mL. In addition, when using AM-EO concentrations up to 40 μg/mL and 80 μg/mL, the level of superoxide anions could be decreased to the normal range, similar to that of the untreated cells (Figure 3A). In over-reactive immune response and autoimmune disease, the presence of superoxide leads to the death of the healthy cells in the inflammatory tissues, and the reduction of superoxide is sometimes beneficial for regulating immune response [30]. Therefore, diminution of superoxide anion production in LPS-stimulated RAW 264.7 macrophages can be ascribed to the anti-inflammatory activity of AM-EO.

We also tested lipid peroxidation through the production of MDA and cellular GSH concentration in LPS-stimulated macrophages to verify the anti-inflammatory activity of AM-EO. The results are shown in Figure 3B,C. The MDA production of AM-EO-treated cells was clearly decreased in a dose-dependent manner when compared with control LPS-treated cells (Figure 3B). The result indicated that AM-EO may successfully repress cellular lipid peroxidation in LPS-stimulated macrophages. In addition, the cellular GSH levels of LPS-stimulated macrophages were obviously decreased in AM-EO treated cells at concentration of 20 μg/mL. Although the suppression effect was not clearly correlated with its concentration (Figure 3C), AM-EO was obviously shown to act as an antioxidant in LPS-stimulated macrophages, thus reducing the levels of cellular GSH.

Generally, the inflammatory response of LPS-stimulated macrophages may damage neighboring cells in addition to the macrophages themselves. To confirm whether AM-EO can protect cells from the damage of the inflammatory response, we analyzed the LPS-induced DNA damage of AM-EO-treated macrophages using a DNA fragmentation analysis. The cells treated with all tested concentrations of AM-EO reveal less DNA laddering than the LPS-treated cells, as shown in Figure 3D. Thus, this dose-dependent effect may help us to understand how AM-EO can effectively suppress LPS-induced apoptosis in RAW 264.7 macrophages (Figure 3D).

### 2.4. The Effect of AM-EO on Superoxide Dismutase (SOD), Catalase (CAT) and Glutathione Peroxidase (GPx) Activities

**Figure 4:**
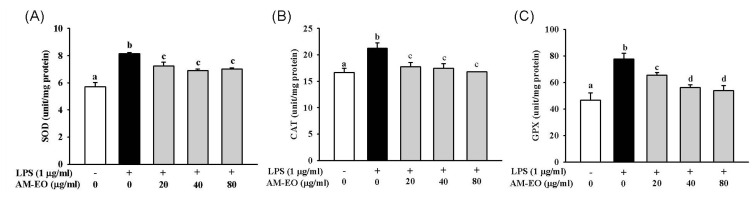
The effect of AM-EO on (**A**) superoxide dismutase (SOD), (**B**) catalase (CAT) and (**C**) glutathione peroxidase (GPx) production by LPS-induced RAW 264.7 macrophages. Each value represents the mean ± SD (*n* = 3). Groups sharing the same superscript letter are not significantly different (*p* > 0.05) as revealed by Dunnett’s *post hoc* tests.

Antioxidant enzymes such as SOD, catalase and GPx play an important function in maintaining the redox homeostasis within cells. Accordingly, these antioxidant enzymes also respond when cells respond to inflammation [31]. In Figure 4, the activities of SOD, CAT and GPx in AM-EO-treated cells were diminished at concentrations ranging from 20 to 80 μg/mL in a dose-dependent manner. In addition, the CAT activity in 80 μg/mL AM-EO-treated cells was nearly the same as that of normal RAW 264.7 macrophages (Figure 4B).

The results of these studies of important antioxidant enzymes demonstrated that the anti-antioxidant activity of AM-EO was not affected by increasing the SOD, CAT and GPx activities in the LPS-stimulated macrophages. Many compounds show antioxidant functions when levels of antioxidant-related enzymes increase [32,33]. However, the activities of antioxidant-related enzymes are down-regulated in some antioxidant-treated cells because the oxidative stress in cells was directly attenuated by the antioxidants [34,35]. Accordingly, combined with the previous results of similar studies, we suggest that AM-EO can repress the oxidative stress and lipid peroxidation of LPS-stimulated macrophages and that the antioxidant activity is not executed via increasing SOD, CAT and GPx activities.

### 2.5. The Effects of AM-EO on the Expression Levels of iNOS, COX-2, TNF-α, IL-6 and HO-1 mRNA

**Figure 5:**
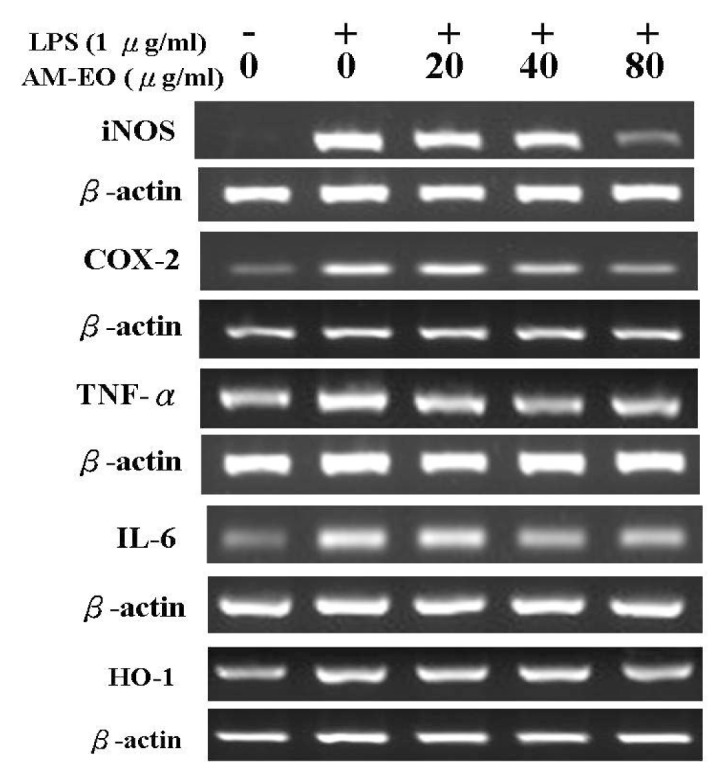
The effect of AM-EO on the mRNA expression levels of iNOS, COX-2, TNF-α, IL-6 and HO-1 in LPS-stimulated RAW 264.7 macrophages.

**Figure 6:**
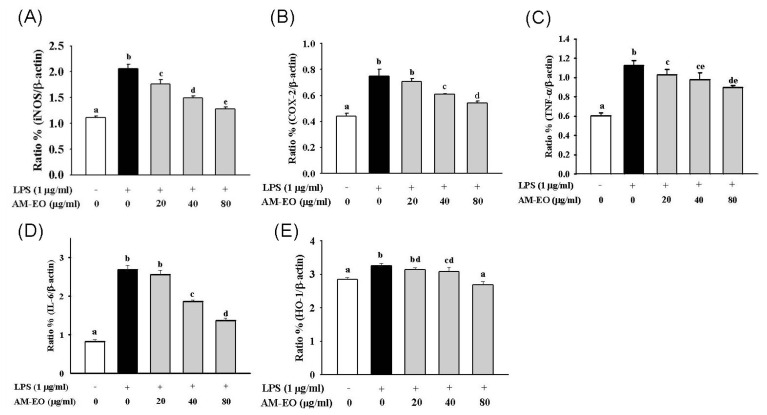
The quantitative mRNA ratio of (**A**) iNOS, (**B**) COX-2, (**C**) TNF-α, (**D**) IL-6 and (**E**) HO-1. Each value represents the mean ± SD (*n* = 3). Groups sharing the same superscript letter are not significantly different (*p* > 0.05) as revealed by Dunnett’s *post hoc* tests.

In macrophages, iNOS, COX-2, TNF-α, IL-6 and HO-1 are the key enzymes or cytokines that participate in inflammatory progression. The activation of iNOS may cause NO accumulation in the cell supernatant while COX-2 (inducible COX) may convert arachidonic acid (AA) to prostaglandins, collectively enhancing the inflammatory response. Additionally, the expression of HO-1 may decrease the level of ROS and suppress the inflammatory response by reducing the function of NO. Additionally, TNF-α and IL-6 are pro-inflammatory cytokines and are highly expressed during the inflammation process [36,37]. Hence, the mRNA expression levels for these key enzymes and cytokines are important indicators for the investigation of anti-inflammatory activity. The specific primers used to determine the expression levels of these mRNAs via RT-PCR are listed in Table 2. The RT-PCR results are shown in Figure 5, and the quantitation of these results is shown in Figure 6.

The results show similar dose-dependent decreases in the mRNA levels of iNOS (Figures 5 and 6A) and COX-2 (Figures 5 and 6B) at all tested concentrations of AM-EO. In the 80 μg/mL AM-EO-treated cells, iNOS and COX-2 mRNA levels are decreased to just slightly higher than those in normal cells. Thus, our results show that AM-EO may protect RAW 264.7 macrophages from the inflammatory response through the inhibition of iNOS and COX-2 expression.

The expression levels of TNF-α and IL-6 mRNA in AM-EO-treated cells are shown in Figures 5 and 6C,D. The mRNA level of TNF-α clearly decreased when the AM-EO concentration was higher than 20 μg/mL. Moreover, the suppression of mRNA expression was correlated with the AM-EO concentrations (Figures 5 and 6C). Similar to the case of TNF-α, the mRNA level of IL-6 was also decreased at every concentration tested in AM-EO-treated cells (Figures 5 and 6D). Unlike TNF-α mRNA expression, IL-6 mRNA expression in AM-EO-treated cells exhibited a significant decrease; at 80 μg/mL AM-EO, the expression of IL-6 mRNA was reduced by approximately 50% when compared with the levels in LPS-stimulated cells.

AM-EO also decreased the mRNA levels of HO-1 in the LPS-stimulated RAW 264.7 macrophages at all concentrations tested (Figures 5 and 6E). However, the decreases in the levels of HO-1 mRNA expression at AM-EO concentrations of 20 and 40 μg/mL were not significant. After the application of 80 μg/mL AM-EO, the HO-1 mRNA expression level was restored to the levels found in normal cells (Figures 5 and 6E). Therefore, the results indicate that the protective effect of AM-EO on LPS-stimulated RAW 264.7 macrophages may be not linked to HO-1. Similar to other antioxidant enzymes, HO-1 expression levels are decreased because AM-EO may inhibit the inflammatory response via its antioxidant activity. Therefore, our results indicate that AM-EO may suppress the LPS-induced inflammatory response of RAW 264.7 macrophages. This suppression was mediated by the down-regulation of iNOS, COX-2, TNF-α and IL-6 expression. Additionally, HO-1 expression is diminished because AM-EO inhibits the inflammatory response through its antioxidant activity.

## 3. Experimental Section

### 3.1. Essential Oil and Cell Line

Steam-distilled essential oil of *Achillea millefolium* L. (AM-EO) was purchased from Australian Botanical Products, Pty Ltd. (Hallam, Victoria, Australia). The murine macrophage cell line RAW 264.7 (BCRC 60001) was obtained from the Bioresource Collection and Research Center (BCRC, Hsinchu, Taiwan) and was used in anti-inflammatory activity assays.

### 3.2. Materials

Fetal bovine serum (FBS), l-glutamine, penicillin-streptomycin, trypsin-ethylenediaminetetraacetic acid (trypsin-EDTA), deoxynucleotide triphosphate (dNTP), oligo(dT), Taq DNA polymerase and Dulbecco’s Modified Eagle Medium (DMEM) medium were purchased from Gibco BRL/Invitrogen (Carlsbad, CA, USA). Lipopolysaccharide (LPS; from *Escherichia coli*, serotype O111: B4), 3-(4,5-dimethylthiazol-2-yl)-2,5-diphenyl tetrazolium bromide (MTT), Griess reagent, sodium nitrite, pyrogallol, nitro blue tetrazolium (NBT), 2-nitrobenzoic acid, nicotinamide adenine dinucleotide phosphate (NADPH), hydrogen peroxide solution (H_2_O_2_), bovine serum albumin (BSA), ethidium bromide, dithiothreitol (DTT), agarose and other chemicals were purchased from Sigma-Aldrich (St. Louis, MO, USA). Deionized distilled water (ddH_2_O) used to prepare solutions and buffers were purified using a Milli-Q system (Millipore, Bedford, MA, USA).

### 3.3. Gas Chromatography and Mass Spectrometry Analysis

GC-MS analyses were carried out on a GCMS-QP-2010 plus Gas chromatograph Mass Spectrometer (Shimadzu, Japan) and using GCMS-solution software (v. 2.50 SU3, Shimadzu, Japan). Compounds were separated on a Forte ID-BPX5 cross-linked 5% phenyl-95% methyl polysiloxane (30 m × 0.25 mm internal diameter (i.d.), film thickness 0.25 μm) capillary column (SGE Analytical Science, Ringwood, Victoria, Australia). The column was maintained at 50 °C for 5 min after injection, then programmed at 5 °C/min to 150 °C, and finally, programmed at 10 °C/min to 300 °C. The injection volume comprised 1.0 μL of pure essential oil with a split ratio of 1:100. Helium was used as the carrier gas at a constant flow-rate of 1.0 mL/min. The injector, transfer line and ion-source temperatures were 250, 230 and 250 °C, respectively. MS detection was performed with an electron impact mode at 70 eV ionization energy and 60 μA ionization current, operating in the full-scan acquisition mode in the 40–350 amu range. Compounds were identified by comparing the retention times and retention indices of the chromatographic peaks with a standard library, National Institute of Standards and Technology (NIST) MS spectral database (version 2005, NIST, Gaithersburg, MD, USA, 2005) and comparing the measured Kovats index (KI) to a homologous series of n-alkanes (C_5_–C_26_).

### 3.4. Cell Culture

RAW 264.7 cells were cultured in DMEM supplemented with 10% FBS, 2 mM l-glutamine and 1% penicillin-streptomycin (100 U/mL penicillin and 100 μg/mL streptomycin). The cells were maintained in a humidified incubator at 37 °C with 5% CO_2_. The cells were sub-cultured every 3–4 days to maintain logarithmic growth and were allowed to grow for 24 h before treatments were applied. The cells were treated with different concentrations of the essential oil (0, 20, 40 and 80 μg/mL) and 1 μg/mL LPS for 20 h.

### 3.5. Cell Viability

RAW 264.7 cells were plated in 12-well plates at a density of 3 × 10^5^ cells/mL. The cells were treated with different concentrations (0, 20, 40 and 80 μg/mL) of essential oil and LPS (l μg/mL) and grown at 37 °C in 5% CO_2_ and 95% air for 20 h. A MTT assay was used to determine cell viability [38].

### 3.6. Nitrite Production

Nitrite was measured as an indicator of NO production after 20 h of essential oil treatment and LPS induction. A 100 μL aliquot of the culture supernatant was plated in a 96-well plate, and an equal amount of Griess reagent (1% sulfanilamide and 0.1% *N*-1-(naphthyl) ethylenediamine dihydrochloride in 2.5% H_3_PO_4_) was added. The plate was then incubated for 5 min, and the absorbance was measured at 540 nm. The amount of NO was calculated using a sodium nitrite standard curve [39].

### 3.7. Measurement of Superoxide Anion Production, Lipid Peroxide and Glutathione (GSH) Levels

RAW 264.7 cells were incubated with various concentrations of essential oil (0, 20, 40 and 80 μg/mL) and LPS (l μg/mL) for 20 h prior to testing the levels of superoxide anions. The superoxide anion measurement, based on the NBT assay, was performed according to the method of Freire *et al*. [40]. For lipid peroxide level measurement, cells were harvested and sonicated in 1 mL of cell lysis buffer containing 1 mM phenylmethylsulfonyl fluoride (PMSF) to obtain a cell homogenate. The thiobarbituric acid reactive substances (TBARS) method was used to estimate cellular malondialdehyde (MDA) levels with a spectrophotometer by measuring the absorbance at 535 nm [41]. Glutathione (GSH) concentration was measured using an enzymatic recycling procedure in which GSH is sequentially oxidized by 2-nitrobenzoic acid and reduced by NADPH in the presence of GSH reductase [42]. The protein content of the cell homogenate was determined based on the Biuret reaction [43] using a BCA kit (Pierce, Rockford, IL, USA) with BSA standards. The MDA and GSH levels in cells are expressed as nanomole per milligram protein.

### 3.8. DNA Fragmentation Assay

RAW 264.7 cells were incubated with various concentrations of essential oil (0, 20, 40 and 80 μg/mL) and LPS (l μg/mL) for 20 h. The cells were then harvested by centrifugation. The DNA was isolated, separated by gel electrophoresis, stained with ethidium bromide and photographed under UV light according to the method of Lu *et al*. [44].

### 3.9. Measurement of Superoxide Dismutase (SOD), Catalase (CAT), Glutathione Peroxidase (GPx) Activity

RAW 264.7 cells were incubated with various concentrations of essential oil (0, 20, 40 and 80 μg/mL) and LPS (l μg/mL) for 20 h. Cells were harvested and sonicated in 1 mL of cell lysis buffer containing 1 mM PMSF to obtain a cell homogenate. SOD activity was determined by spectrophotometry based on the absorbance readings obtained at 325 nm, which indicate the SOD-mediated decrease in the rate of pyrogallol autoxidation under alkaline conditions [45]. A unit of SOD activity was defined as the amount of enzyme that inhibited the rate of pyrogallol oxidation. Catalase activity was analyzed by following the decrease in absorbance of H_2_O_2_ at 240 nm. One unit of catalase was defined as the amount of enzyme that decomposed 1.0 μM of H_2_O_2_ per minute [46]. For glutathione peroxidase (GPx) activity, one unit of GPx was defined as the amount of enzyme that oxidized 1 nM of NADPH per minute based on the absorbance readings obtained at 340 nm [47]. The specific activities of SOD, CAT and GPx are expressed as unit/mg protein.

### 3.10. Measurement of Messenger RNA (mRNA) Levels of Inducible nitric Oxide Synthase (iNOS), Cyclooxygenase-2 (COX-2), Tumour Necrosis Factor-α (TNF-α), Interleukin-6 (IL-6) and Heme Oxygenase-1 (HO-1)

RAW 264.7 cells were incubated with various concentrations of essential oil (0, 20, 40 and 80 μg/mL) and LPS (l μg/mL) for 20 h, and the total RNA was extracted using the Qiagen RNeasy Mini Kit (Qiagen, Inc, Valencia, CA, USA). Briefly, a reverse transcription (RT) reaction was performed using 5 μg of total RNA, 1 μL of oligo(dT), 1 μL of dNTP mix (10 mM) and up to 12 μL of ddH_2_O. The mixture was heated for 5 min at 65 °C and quickly chilled on ice. Subsequently, 4 μL of first strand buffer, 2 μL of 0.1 M DTT and 1 μL of RNAseOUT were added to the mixture. The mixture was incubated at 37 °C for 2 min, and 1 μL of M-MLV reverse transcriptase was added. The reaction was stopped by heating the solution to 70 °C for 15 min. A 1 μL aliquot of cDNA mixture was used in the subsequent enzymatic amplification. Polymerase chain reaction (PCR) was performed using 1.5 mM MgCl_2_, 0.2 mM dNTP, 2.5 units of Taq DNA polymerase and 0.1 μM each of the primers targeting iNOS, COX-2, TNF-α, IL-6 and HO-1 (Table 2). The amplified products were separated in 2% agarose gel in Tris-borate-EDTA (TBE) buffer and stained with ethidium bromide [48].

### 3.11. Statistical Analysis

All assays were conducted at least three times with three different sample preparations. All data are expressed as the mean ± standard deviation (SD). An analysis of variance was performed using SPSS version 16.0 (SPSS Inc., Chicago, IL, USA, 2007). A one-way ANOVA and Dunnett’s *post hoc* test were used for these analyses, and *p* < 0.05 was considered to be statistically significant.

## 4. Conclusions

In summary, 19 compounds had been identified in AM-EO. Among them, artemisia ketone (14.92%), camphor (11.64%), linalyl acetate (11.51%) and 1,8-cineole (10.15%) are the main components. Obvious inhibition of inflammatory responses and oxidative stress of LPS-stimulated RAW 264.7 macrophages was found when AM-EO was present in the medium. This antioxidant activity is not mediated by increasing SOD, CAT and GPx activities, but by the down-regulation of iNOS, COX-2, TNF-α and IL-6 expression. HO-1 expression is also decreased because AM-EO reduces the inflammatory response, mainly due to its own antioxidant activity. The effects of AM-EO infer that such natural volatilized oils provide, not only flavor, but also possess biological activities. Therefore, due to these antioxidant and anti-inflammatory activities, the essential oil volatilized from *A. millefolium* could be used in many applications in the future, including utilization as a functional ingredient in health foods or as a drug for treating inflammatory related diseases.

## Figures and Tables

**Table 1:** Chemical composition of *Achillea millefolium* L. essential oil.

No.	Compounds	KI a	Peak area (%)
01	Camphene	929	1.79
02	alpha-Pinene	939	1.18
03	beta-Pinene	943	2.38
04	Yomogi alcohol	1002	6.36
05	o-Cymene	1021	5.26
06	D-Limonene	1042	7.39
**07**	**1,8-Cineole**	**1059**	**10.15**
**08**	**Artemisia ketone**	**1065**	**14.92**
09	Artemisia alcohol	1068	2.41
10	Linalool	1082	6.55
11	Thujone	1097	1.68
**12**	**Camphor**	**1121**	**11.64**
13	Borneol	1138	5.37
14	Terpinenol-4-ol	1139	3.69
15	(*Z*)-Chrysanthenyl acetate	1276	1.44
**16**	**Linalyl acetate**	**1279**	**11.51**
17	Caryophyllene	1494	1.83
18	Germacrene D	1515	2.76
19	Viridiflorol	1530	1.69
	Monoterpene hydrocarbons		18.00
	Oxygenated monoterpenes		75.72
	Sesquiterpene hydrocarbons		4.59
	Oxygenated sesquiterpenes		1.69
	Total identified		100.00

a Kovats index.

**Table 2:** Primer sequences used for RT-PCR in this study.

Target	Type	Sequences
β-actin	Sense	5′-TGGAATCCTGTGGCATCCATGAAAC-3′
	Anti-sense	5′-TAAAACGCAGCTCAGTAACAGTCCG-3′
iNOS	Sense	5′-AGACTGGATTTGGCTGGTCCCTCC-3′
	Anti-sense	5′-AGAACTGAGGGTACATGCTGGAGCC-3′
COX-2	Sense	5′-GGAGAGACTATCAAGATAGT-3′
	Anti-sense	5′-ATGGTCAGTAGACTTTTACA-3′
TNF-α	Sense	5′-GGCAGGTCTACTTTGGAGTCATTGC-3′
	Anti-sense	5′-ACATTCGAGGCTCCAGTGAATTCGG-3′
IL-6	Sense	5′-GAGGATACCACTCCCAACAGA-3′
	Anti-sense	5′-AAGTGCATCATCGTTGTTCATACA-3′
HO-1	Sense	5′-TGAAGGAGGCCACCAAGGAGG-3′
	Anti-sense	5′-AGAGGTCACCCAGGTAGCGGG-3′
